# Study on the Antitumor Effect and Glycolysis of Andrographolide in Anaplastic Thyroid Carcinoma

**DOI:** 10.1155/2021/5526581

**Published:** 2021-07-14

**Authors:** Ke Yang, Ke Wu, Jianguo Feng, Ling Yutian, Xin Zhu, Dong Xu

**Affiliations:** ^1^The Cancer Hospital of the University of Chinese Academy of Sciences (Zhejiang Cancer Hospital), Institute of Basic Medicine and Cancer (IBMC), Chinese Academy of Sciences, Hangzhou, Zhejiang 310022, China; ^2^Key Laboratory of Head & Neck Cancer Translational Research of Zhejiang Province, Hangzhou, Zhejiang 310022, China

## Abstract

**Objective:**

To investigate the antitumor effect of andrographolide on the ATC cell lines 8505C and CAL62 and to explore the possible mechanism of the effect.

**Methods:**

CCK8 and colony formation assays were performed to detect proliferation. Cell migration was tested by scratch assay. Annexin V/PI staining was used to detect cell apoptosis and cell cycle. Glucose and lactic acid kits were carried out to evaluate the glycolysis level after andrographolide treatment. Western blot was used to detect the changes in the apoptosis-related proteins and glycolysis-related enzymes in both 8505C and CAL62 cells.

**Results:**

Treatment with 60 *μ*M andrographolide had significant effects on 8505C and CAL62, including inhibition of proliferation, inhibition of migration, arrest of the cell cycle, promotion of apoptosis, and inhibition of glycolysis.

**Conclusion:**

Andrographolide has an antitumor effect and can significantly affect glycolysis in ATC cells.

## 1. Introduction

Thyroid carcinoma is a common malignancy in the endocrine system. In recent years, thyroid carcinoma has become one of the fastest-growing malignant tumors and has received widespread attention [1]. According to tumor origins and differences in differentiation, thyroid carcinoma can be divided into papillary thyroid carcinoma (PTC), follicular thyroid carcinoma (FTC), medullary thyroid carcinoma (MTC), and anaplastic thyroid carcinoma (ATC). ATC is a highly malignant tumor and is prone to lymph node and distant metastases, and the fatality rate can be as high as 50% of all types of thyroid carcinoma [2, 3]. Moreover, the course of ATC progresses rapidly, and the median survival time of confirmed patients is less than 6 months [4, 5], while the survival rate within one year is only 10% [6]. In short, ATC has a high degree of malignancy and poor prognosis, and no optimal treatment has been established so far. The effects of traditional surgery, chemotherapy, and radiotherapy are not ideal. In recent years, great progress has been made in the understanding of genomic profiles of ATC, which provides effective molecular targets for the treatment of ATC. Specifically, various gene mutations in ATC have been proved, such as *p53* mutation, *TERT* promoter mutation, *BRAF* mutation, *RAS* mutation, and *PI3KCA* mutation [7, 8]. The main signal transduction pathways involved in ATC carcinogenesis are *RAS/RAF/MEK* and *PI3K/AKT/mTOR* pathway [9]. Based on these findings, the targeted therapy of ATC has also made some development. Current studies have proved that the combination therapies including radiotherapy, chemotherapy, and the targeted therapies might control the progress and improve the prognosis of ATC patients.

**Figure 1:**
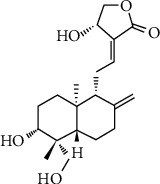
Andrographolide's chemical structure formula.

For the past few years, increasing attention has been given to some natural plants, and research on these plants has become a hot spot. Andrographolide is a natural diterpene lactone isolated and identified from *Andrographis paniculata*. The chemical structure formula of andrographolide is shown in Figure 1. Modern pharmacological studies have shown that andrographolide and its derivatives have some effects, including anti-inflammatory [10, 11], antiviral [12], antitumor [13], hepatoprotective and choleretic [14], immune regulation [15], and other effects. Jada et al. [16] reported the semisynthesis of andrographolide derivatives and their anticancer activities in vitro, which might be associated with cell cycle progression; ADR reduced VSMC cell proliferation by inducing apoptosis through ceramidep47phox-ROS signaling cascade [17]. Furthermore, it has been studied that andrographolide-induced cell proliferation decrease may be attributed to the inhibition of fatty acid synthesis, iron uptake, protein synthesis, and FLT3 signaling in MV4-11 cells [18]. In esophageal cancer, *Andrographis paniculata* has also been reported to exert an antitumor effect and is related to multiple signaling pathways including WNT, TGF-*β*, MAPK, and ErbB pathways [19]. In addition, some studies have found that andrographolide has obvious inhibitory effects on lung, liver, gastric, esophageal, and breast cancers, among others [20], showing good antitumor activity of andrographolide. However, there is no relevant research about andrographolide in ATC cells. Therefore, there is certain research significance in exploring the antitumor activity of andrographolide on ATC cells.

Glycolysis is a ubiquitous energy metabolism process in organisms and refers to the process in which glucose undergoes a series of enzymatic reactions to generate pyruvate and then reduces to lactic acid under conditions of insufficient oxygen. In recent years, an increasing number of studies have found that an anticancer effect can be achieved by inhibiting glycolysis [21, 22]. Intriguingly, andrographolide has been shown [23] to affect glucose metabolism by targeting Hypoxia-Inducible Factor 1a (HIF-1a), Nuclear Factor Kappa B (NF-kB), MAPK-Src, AP-1, JAK/STAT, Nrf2/keap1, and AMP-Activated Protein Kinase (AMPK) pathway, which may contribute to the effect of andrographolide on inflammation and cancer progression. Glycolysis also plays a very important role in thyroid carcinoma. Suh et al. found that glycolysis is closely related to the differentiation of thyroid tumors. The glycolytic characteristics of thyroid cancer may be a prognostic indicator suggesting the risk stratification of thyroid cancer [24], and according to a study conducted by Hébrant et al., ATC showed a stronger glycolytic performance, which suggested that the significance and value of glycolysis in ATC are more important than those in PTC [25]. Meanwhile, studies have shown that inhibiting glycolysis in ATC cells can make ATC cells more sensitive to radiotherapy and chemotherapy [26, 27]. In summary, glycolysis plays a significant role in the occurrence and development of thyroid cancer, especially in ATC. Therefore, the study of andrographolide on glycolysis in ATC cells is conducive to the mechanism of andrographolide in ATCs and can provide certain laboratory guidance for the clinical treatment of ATC.

## 2. Materials and Methods

1. Main reagents and instruments: 8505C and CAL62 cells were all obtained from the Key Laboratory of Head and Neck Cancer Translational Research of Zhejiang Cancer Hospital. Andrographolide (TargetMol), RPMI Medium Modified (HyClone), fetal bovine serum (Gibco), PBS (Gibco), pancreatin (SEGMA), PVDF membrane (Shanghai Beyotime Biotechnology Co., Ltd.), SDS-PAGE gel rapid preparation kit (Shanghai Beyotime Biotechnology Co., Ltd.), milk powder (Biofroxx), 20xTBST (Solarbio), CCK8 (ApexBio), Annexin V-EGFP Apoptosis Detection Kit (Hangzhou Lianke Biotechnology Co., Ltd.), cycle detection kit (Hangzhou LinkTech Biotechnology Co., Ltd.), BCA protein concentration determination kit (Shanghai Beyotime Biotechnology Co., Ltd.), RIPA Lysis Buffer (Shanghai Beyotime Biotechnology Co., Ltd.), PMSF (Shanghai Beyotime Biotechnology Co., Ltd.), a lactic acid detection kit (Nanjing Jiancheng Biological Engineering Institute Co., Ltd.), and a glucose detection kit (Shanghai Rongsheng Biopharmaceutical Industry Co., Ltd.) were used in this research, and all of the antibodies were purchased from Proteintech. A carbon dioxide incubator (Thermo 3100), biological safety cabinet (Thermo Electron 1287), flow cytometer (BD FACSV), chemiluminescence detector (Thermo Varioskan Flash), electrophoresis instrument (Bio-Rad 1645050), and automatic gel image analysis system (Bio-Rad ChemDoc XPS+) were also included.
2. Cell culture: 8505C and CAL62 cells were placed in a 37°C, 5% CO_2_ incubator for culture. The culture medium was 1640 medium containing 10% fetal bovine serum. When the cell growth confluence reached 80%–90%, trypsinization was used for subculture.
3. Cell viability test: two strains of 8505C and CAL62 cells were inoculated in a 96-well plate at 3000 cells per well. After 24 hours, six groups consisting of a control group and andrographolide groups (15, 30, 45, 60, and 75 *μ*M) were set up, and fresh culture medium was added to each group. Each group had 3 auxiliary holes. After 48 h of drug treatment, 10 *μ*L of CCK8 was added to each well and incubated at 37°C for 4 h, and the absorbance at 450 nm was measured using a microplate reader.
4. Colony formation experiment: 8505C and CAL62 cells were inoculated in a six-well plate at 3000 cells per well. Six groups consisting of a control group and andrographolide groups (15, 30, 45, 60, and 75 *μ*M) were set. After overnight adherence, the corresponding concentration of andrographolide was used for 48 hours, the medium was changed, and the culture was continued for 7 days. After 7 days, the supernatant was discarded, and the cells were washed twice with PBS, fixed with methanol for 15 minutes in each well, stained with 300 *μ*L of crystal violet for 30 minutes, and washed twice with PBS, and the number of colonies was counted.
5. Wound healing: two ATC cell lines (8505C and CAL62) were seeded into a six-well plate with a density of 300000 cells/well. Then, four groups were set up: a control group and three andrographolide (30, 45, and 60 *μ*M) groups. After adherence to the wall overnight and scratch injury, pictures were taken, and the same position was recorded at 0, 24, 48, and 72 h to compare the healing of scratches and to investigate the effect of andrographolide on the migration ability of ATC cells.
6. Flow cytometry detection of apoptosis: two ATCs (8505C and CAL62) were plated on a six-well plate with a density of 100000 cells/well. And a control group and five groups of andrographolide (15, 30, 45, 60, and 75 *μ*M) were set up. After overnight adherence, the cells were treated with the corresponding concentration of andrographolide for 48 hours, collected, washed twice with precooled PBS, stained with Annexin V/PI, and the percentage of apoptosis was measured by flow cytometry.
7. Cell cycle analysis by flow cytometry: two ATCs (8505C and CAL62) were plated on a six-well plate with a density of 100000 cells/well. And two groups of cells, a control group and an andrographolide group (60 *μ*M), were set up. After overnight adherence, the corresponding concentration of andrographolide was applied in andrographolide for 48 hours of treatment, and the cells were collected and washed twice with precooled PBS. After PI staining, the cell cycle distribution was detected by flow cytometry.
8. Western blot detection of apoptosis-, cell cycle-, and glycolysis-related proteins: two types of ATC cells (8505C and CAL62) were plated in six-well plates, and two groups, a control group and an andrographolide group (60 *μ*M), were set up and allowed to adhere to the walls overnight. The cells were treated with andrographolide for 48 hours and collected. Western blot was used to detect the changes in the apoptosis-related proteins and glycolysis-related enzymes. The influences of the proteins Bcl-2, Bcl-XL, PARP, cleaved PARP, Caspase-3, cleaved Caspase-3, HK2, PKM2, PFKM, LDHA, and GLUT1 were evaluated.
9. Determination of glucose and lactic acid contents in the supernatant of ATC cells after andrographolide administration: two types of ATC cells (8505C and CAL62) were plated in 96-well plates with a density of 3000 cells/well. And six groups, a control group and andrographolide groups (15, 30, 45, 60, and 75 *μ*M), were established. After allowing the cells to adhere to the walls overnight, they were treated with andrographolide at the corresponding concentration. The supernatant was collected at the three time points of 0, 24, and 48 h, and the glucose and lactic acid in the supernatant were detected with glucose and lactic acid kits.
10. Statistical methods: all experiments were repeated three times independently, and the data are expressed as *x* ± *s* and were analyzed by SPSS 17.0 software. The data were tested by *t* test, and *P* < 0.05 was considered statistically significant. *P* < 0.05 is indicated by ^*∗*^, *P* < 0.01 is indicated by ^*∗∗*^, and *P* < 0.001 is indicated by ^*∗∗∗*^. The smaller the *P* value (the more asterisks) was, the more significant the difference was. GraphPad Prism 7 was used in this study to draw statistical charts.

## 3. Results

### 3.1. Changes in the Proliferation Abilities of 8505C and CAL62 ATC Cells after Andrographolide Treatment

**Figure 2:**
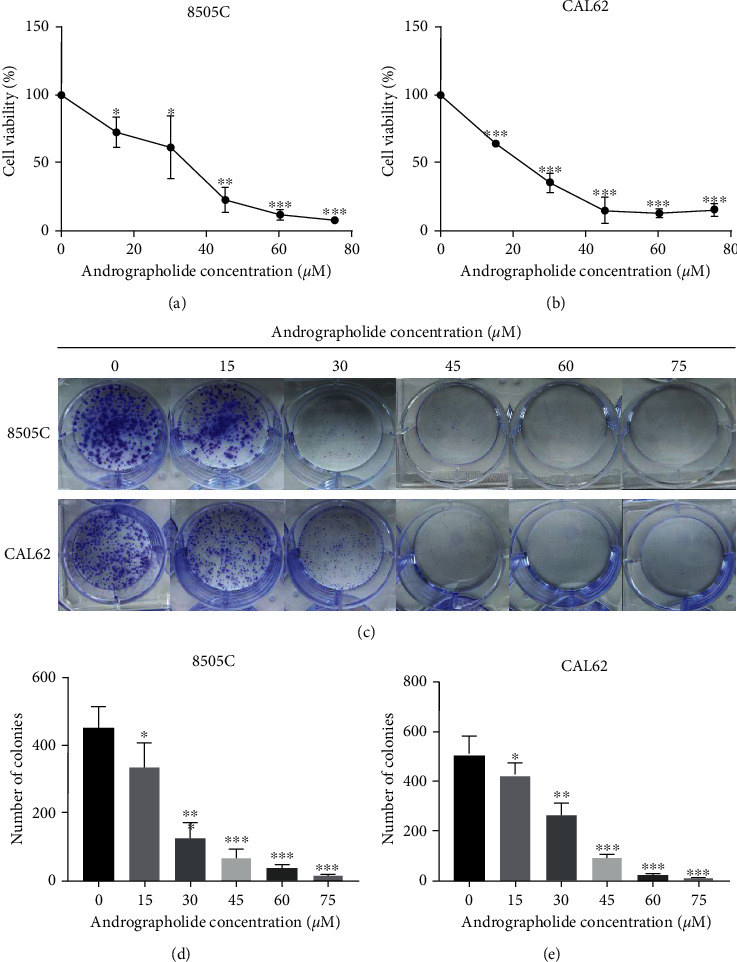
Effects of andrographolide on the proliferation of 8505C and CAL62 ATC cells. (a) Changes in the viability of 8505C cells under different concentrations of andrographolide. (b) Changes in the viability of CAL62 cells under different concentrations of andrographolide. (c) Colony formation of 8505C and CAL62 cells under different concentrations of andrographolide. (d) Statistics of 8505C cell colony formation under the actions of different concentrations of andrographolide. (e) Statistics of CAL62 cell colony formation under the actions of different concentrations of andrographolide.

The cell viability results showed that after 48 hours of andrographolide treatment, the viability levels of 8505C and CAL62 ATC cells were significantly (*P* < 0.001) inhibited (Figures 2(a) and 2(b)), and the results of colony formation experiments showed that andrographolide treatment significantly (*P* < 0.001) inhibited 8505C and CAL62 after 48 hours. The colony-forming ability of CAL62 is shown in Figures 2(c)–2(e).

### 3.2. Changes in the Migration Ability of 8505C and CAL62 ATC Cells after Andrographolide Treatment

**Figure 3:**
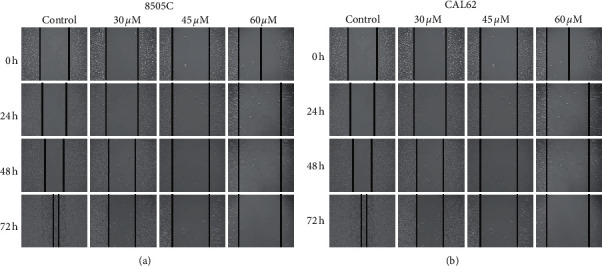
Effects of andrographolide on the migration ability of 8505C and CAL 62 cells. (a) The effect of andrographolide on the migration ability of 8505C cells. (b) The effect of andrographolide on the migration ability of CAL62 cells.

Wound healing assays showed that after 48 hours of andrographolide treatment, the migration ability of 8505C and CAL62 ATC cells was significantly (*P* < 0.01) inhibited (Figures 3(a) and 3(b)).

### 3.3. Changes in the Cell Cycle of 8505C and CAL62 ATC Cells after Andrographolide Treatment

**Figure 4:**
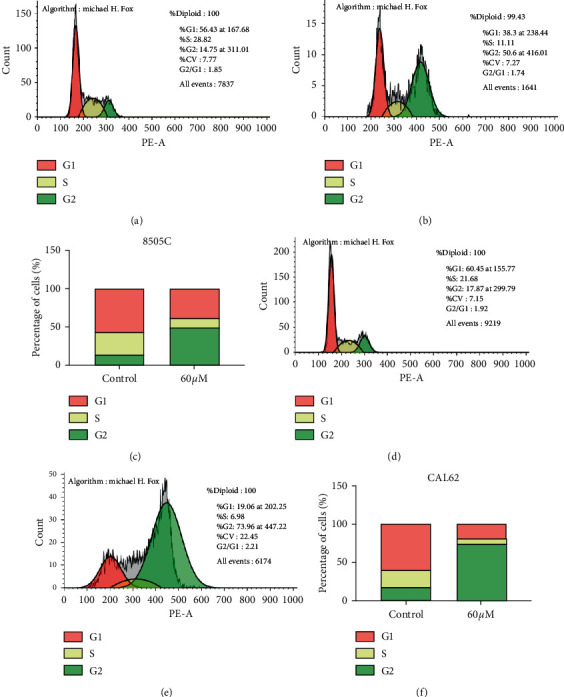
Effect of andrographolide on the cell cycle in 8505C and CAL62 ATC cells. (a) Cell cycle distribution of 8505C cells. (b) Cell cycle distribution of 8505C cells after andrographolide treatment. (c) Cell cycle distribution statistics of 8505C cells after treatment of 8505C cells with andrographolide. (d) Cell cycle distribution of CAL62 cells. (e) Cell cycle distribution of CAL62 cells after andrographolide treatment. (f) Cell cycle distribution statistics of CAL62 cells after the actions of CAL62 and andrographolide.

The cell cycle results showed that after the action of andrographolide, cells in S phase decreased, cells in G1 phase decreased, and cells in G2 phase increased (*P* < 0.01) in 8505C and CAL62 ATC cells. These results suggested that andrographolide could cause ATC cell cycle arrest (Figures 4(a)–4(f)).

### 3.4. Changes in Apoptosis in 8505C and CAL62 ATC Cells after Andrographolide Treatment

**Figure 5:**
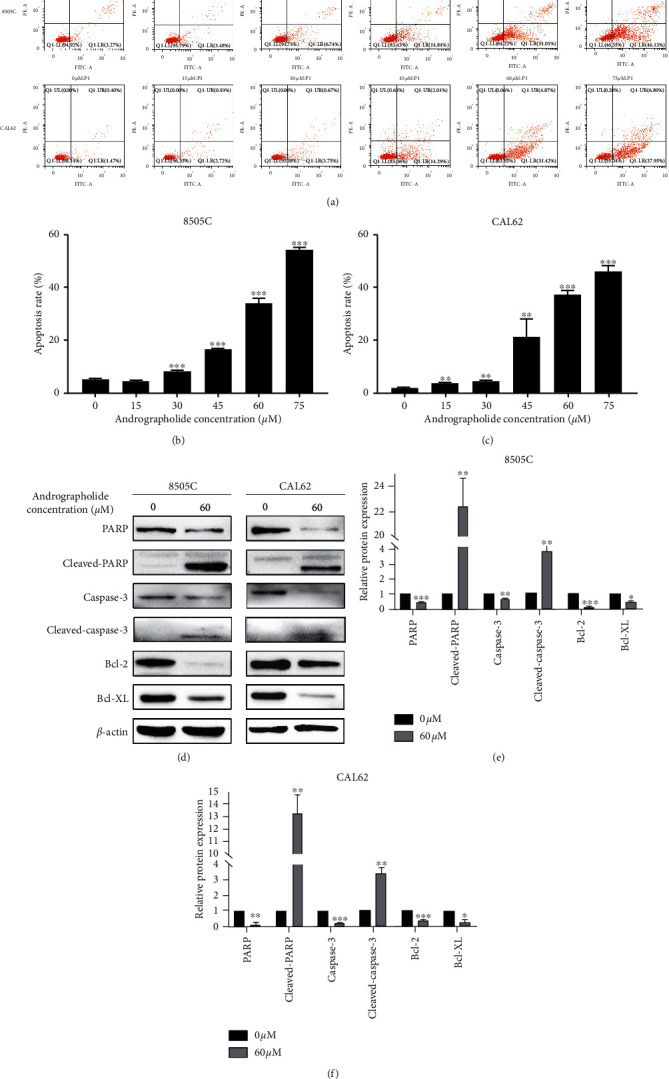
Effects of andrographolide on the apoptosis of 8505C and CAL62 ATC cells. (a) Effects of different concentrations of andrographolide on the apoptosis rates of 8505C and CAL62 cells. (b) Changes in the 8505C apoptosis rate under the action of different concentrations of andrographolide. (c) Changes in the CAL62 apoptosis rate under the action of different concentrations of andrographolide. (d) WB of apoptosis-related proteins in andrographolide-treated 8505C and CAL62 cells after the action of lactone. (e) Apoptosis-related protein changes in 8505C cells after andrographolide treatment. (f) Apoptosis-related protein changes in CAL62 cells after andrographolide treatment.

Apoptosis results showed that after 48 hours of andrographolide treatment, the apoptosis rates of 8505C and CAL62 ATC cells increased significantly (*P* < 0.001). In the 75 *μ*M andrographolide group, the total apoptosis rates in 8505C and CAL62 cells reached 53.65% and 44.96%, respectively (Figures 5(a)–5(c)). After 48 hours of treatment with andrographolide, the apoptosis-related proteins Bcl-2, Bcl-XL, PARP, and Caspase-3 in ATC 8505C and CAL62 cells were downregulated, and cleaved-PARP and cleaved-Caspase-3 were upregulated (Figures 5(d)–5(f)).

### 3.5. Changes in the Glycolysis Levels of 8505C and CAL62 ATC Cells after the Action of Andrographolide

**Figure 6:**
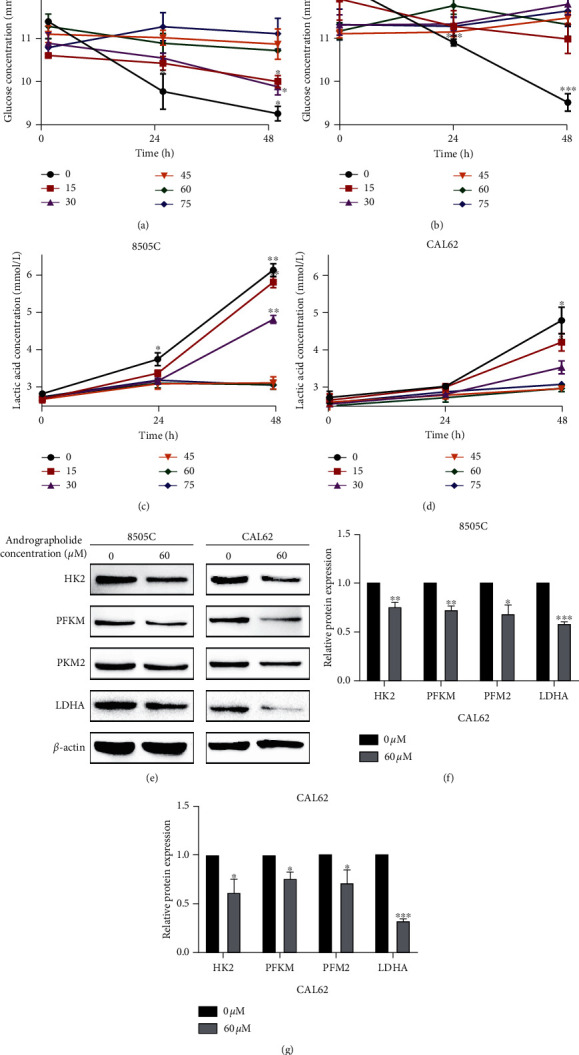
Effects of andrographolide on glycolysis in 8505C and CAL62 ATC cells. (a) Changes in glucose concentration in the supernatant of 8505C cells with time after different concentrations of andrographolide. (b) Changes in glucose concentration in the supernatant of CAL62 cells with time after different concentrations of andrographolide. (c) Effects of different concentrations of andrographolide and changes in the concentration of lactic acid in the supernatant of cells after 8505C with time. (d) Changes in the concentration of lactic acid in the supernatant of CAL62 cells with time after different concentrations of andrographolide. (e) Effects on the expression of glycolysis-related proteins in 8505C and CAL62 cells after andrographolide treatment. (f) Analysis of changes in glycolysis-related proteins in 8505C cells after andrographolide treatment. (g) Analysis of changes in glycolysis-related proteins in CAL62 cells after andrographolide treatment.

After treated with andrographolide for 0, 24, and 48 h, the 8505C and CAL62 cell supernatants were used to determine the glucose and lactic acid contents. The results showed that when the concentration of andrographolide was 0, 15, or 30 *μ*M, the concentrations of glucose and lactic acid in the supernatant of 8505C cells changed significantly (*P* < 0.01) over time, while when the concentration of andrographolide was 45, 60, or 75 *μ*M, the concentrations of glucose and lactic acid in the supernatant of 8505C cells remained essentially unchanged over time. When the concentration of andrographolide was 0 or 15 *μ*M, the concentrations of glucose and lactic acid in the supernatants of CAL62 cells changed significantly (*P* < 0.05) over time, while when the concentration of andrographolide was 30, 45, 60, or 75 *μ*M, the concentrations of glucose and lactate in the supernatant of CAL62 cells remained essentially unchanged over time (Figures 6(a)–6(d)). After 48 hours of andrographolide treatment, the glycolysis-related proteins HK2, PKM2, PFKM, and LDHA in ATC 8505C and CAL62 cells were significantly downregulated (*P* < 0.05) (Figures 6(e)–6(g)).

## 4. Discussion

It has been reported [28] that the mechanism by which andrographolide inhibits tumor cell growth includes inhibition of proliferation, arrest of the cycle, and induction of apoptosis. Liu et al. found that andrographolide could inhibit the proliferation and cell cycle of the human hepatoma cell line MHCC97H and has a certain antitumor activity on liver cancer [29]. Shi et al. showed that andrographolide could inhibit the migration of human colon cancer cells and block the cell cycle to achieve a certain antitumor effect [30]. The results of the present study showed that the cell viability and colony-forming ability of ATC cells were significantly inhibited by andrographolide, indicating that andrographolide can inhibit the proliferation of ATC cells. Meanwhile, the number of cells in each phase of the ATC cell cycle changed significantly after the action of andrographolide. Among them, S phase cells decreased, G1 phase cells decreased, and G2 phase cells increased, indicating that andrographolide can inhibit the cell cycle in G2/M phase. Cell proliferation is achieved through DNA replication and protein synthesis in the cell cycle. The cycle-blocking effect of andrographolide further reflects the inhibitory effect on ATC cell proliferation. After the action of andrographolide, the wound-healing ability of ATCs was significantly reduced, suggesting that andrographolide can inhibit the migration of ATCs. The above results proved that andrographolide could significantly inhibit the proliferation and migration of ATCs and produce cycle-inhibiting effects, indicating that andrographolide has good antitumor activity on ATCs.

Furthermore, the results showed that andrographolide could cause significant apoptosis of ATC cells. In the process of cell apoptosis, the Bcl-2 and Caspase families play vital roles. The Bcl-2 family is an important regulator of mitochondrial apoptosis. Bcl-2 and Bcl-XL are the anti-apoptotic proteins. The results of this study showed that after andrographolide treatment, Bcl-2 and Bcl-XL were downregulated in ATC cells, indicating that andrographolide initiated the mitochondrial apoptosis program. Meanwhile, Caspase activation is one of the most critical links in the mechanism of apoptosis. Among them, Caspase-3 is the main effector Caspase and the key executor of apoptosis. The appearance of apoptosis signals can cause Caspase-3 to be cleaved and activated to cleaved-Caspase-3 under the action of a variety of proteolytic enzymes [31]. Cleaved-Caspase-3 can cleave the downstream protein PARP, which is cleaved into cleaved-PARP [32]. PARP, or poly (ADP-ribose) polymerase, is an enzyme closely related to DNA repair. Cleaved-PARP is the final executor of mitochondrial apoptosis, which ultimately leads to mitochondrial apoptosis [33]. The results of this study indicated that andrographolide could significantly downregulate the anti-apoptotic proteins Bcl-2 and Bcl-XL, upregulate the expression of cleaved-Caspase-3, and significantly induce the production of cleaved-PARP, suggesting that andrographolide can induce apoptosis through the Caspase-3/PARP pathway.

Under aerobic conditions, normal cells in the body obtain adenosine triphosphate (ATP) through aerobic oxidation, and glycolysis is inhibited. However, in many malignant tumors, even if the oxygen supply is sufficient, glycolysis is very active [34]. Glycolysis is first carried out by glucose transporters (GLUTs) to transport glucose into cells. Hexokinase (HK) produces glucose-6-phosphate by phosphorylation and then undergoes phosphate hexose isomerase and phosphofructokinase (PFK). After a series of enzyme actions, it is converted into pyruvate. Pyruvate is converted into lactic acid by lactate dehydrogenase (LDHA) under hypoxic conditions, and lactic acid can be transported to the outside of the cell via a monocarboxylic acid transporter. Glucose is the raw material of the glycolysis pathway, and lactic acid is the product of the glycolysis pathway. The glycolysis pathway of tumor cells consumes a large amount of glucose and produces a large amount of lactic acid at the same time. The results of this study showed that after a certain concentration of andrographolide, the content of glucose and lactic acid in the supernatant of ATC cells remained essentially unchanged from 0 to 48 h, demonstrating that under the action of andrographolide, the glucose in the supernatant of ATC cells is no longer consumed, and the lactic acid content is no longer increased, suggesting that andrographolide can inhibit glycolysis. Moreover, the results of this study showed that after treatment with andrographolide, the expression levels of the glycolysis-related proteins HK2, PKM2, PFKM, and LDHA in 8505C and CAL62 thyroid cells decreased, further proving that andrographolide can inhibit glycolysis in ATC tumor cells.

## 5. Conclusion

Except for andrographolide, many other natural drugs have been proved to inhibit tumor progression [35, 36], which indicated that phytotherapeutics were a major source of novel potential compounds useful in cancer therapy. Studies on thyroid cancer have also confirmed the anticancer activity of plant natural products both in basic research and clinical trials, suggesting that thyroid cancer may be an excellent indication for natural compounds [37]. It can be deduced from the present study that andrographolide might exert antitumor effects by inhibiting glycolysis in ATC cells. Based on the critical role of glycolysis in ATC development and treatment [24, 26], the results of this study reinforce andrographolide as an effective candidate drug for the future treatment of ATC with great research significance and clinical transformation value.

## Data Availability

The data used to support the findings of this study are available from the corresponding author upon request.
